# Preface

**DOI:** 10.2174/1573403X2101250107140613

**Published:** 2025-01-07

**Authors:** Tong Liu

**Affiliations:** 1 Department of Cardiology, Tianjin Institute of Cardiology, Second Hospital of Tianjin Medical University, Tianjin, 300211, People’s Republic of China

Current Cardiology Reviews is an esteemed peer-reviewed journal that focuses on disseminating novel and significant advancements in the field of cardiovascular physiology and pathophysiology. It encompasses a broad spectrum of topics, including but not limited to preventive strategies, diagnostic methodologies, and therapeutic interventions for cardiovascular diseases. Over the years, it has evolved into a reputable international platform that consistently highlights cutting-edge research and developments within the realm of cardiology. The journal serves as a vital resource for cardiologists, researchers, and healthcare professionals by providing comprehensive insights into the latest clinical practices, translational research, and emerging trends in cardiovascular medicine.

This issue of Current Cardiology Reviews presents a collection of high-quality review articles that delve into the detection, prevention, and therapeutic strategies for a range of cardiovascular diseases. The development described in this issue provides comprehensive insights into the latest clinical practices, translational research, and emerging trends in cardiovascular medicine. 

Here, I extend my sincere gratitude to all the authors whose dedication and expertise have contributed to the articles featured in this issue. I am also sincerely grateful to the associate and section editors, as well as the editorial board members, for their constant support and guidance.

I am excited to welcome contributions from worldwide researchers and trust that this articles of this issue will be both insightful and engaging for our readers.

